# Roles of APOBEC3 in hepatitis B virus (HBV) infection and hepatocarcinogenesis

**DOI:** 10.1080/21655979.2021.1931640

**Published:** 2021-05-27

**Authors:** Yuan Zhang, Xiaorong Chen, Yajuan Cao, Zongguo Yang

**Affiliations:** a Department of Integrative Medicine, Shanghai Public Health Clinical Center, Fudan University, Shanghai, China; b Central Laboratory, Shanghai Pulmonary HospitalSchool of Medicine, Tongji University School of Medicine, Shanghai, China; c Clinical Translation Research Center, Shanghai Pulmonary Hospital, Tongji University School of Medicine, Shanghai, China

**Keywords:** APOBEC3, Hepatitis B virus, Cancer, Hepatocellular carcinoma

## Abstract

APOBEC3 (A3) cytidine deaminases inhibit hepatitis B virus (HBV) infection and play vital roles in maintaining a variety of biochemical processes, including the regulation of protein expression and innate immunity. Emerging evidence indicates that the deaminated deoxycytidine biochemical activity of A3 proteins in single-stranded DNA makes them a double-edged sword. These enzymes can cause cellular genetic mutations at replication forks or within transcription bubbles, depending on the physiological state of the cell and the phase of the cell cycle. Under pathological conditions, aberrant expression of A3 genes with improper deaminase activity regulation may threaten genomic stability and eventually lead to cancer development. This review attempted to summarize the antiviral activities and underlying mechanisms of A3 editing enzymes in HBV infections. Moreover, the correlations between A3 genes and hepatocarcinogenesis were also elucidated.

## Graphical abstract

**Figure:**
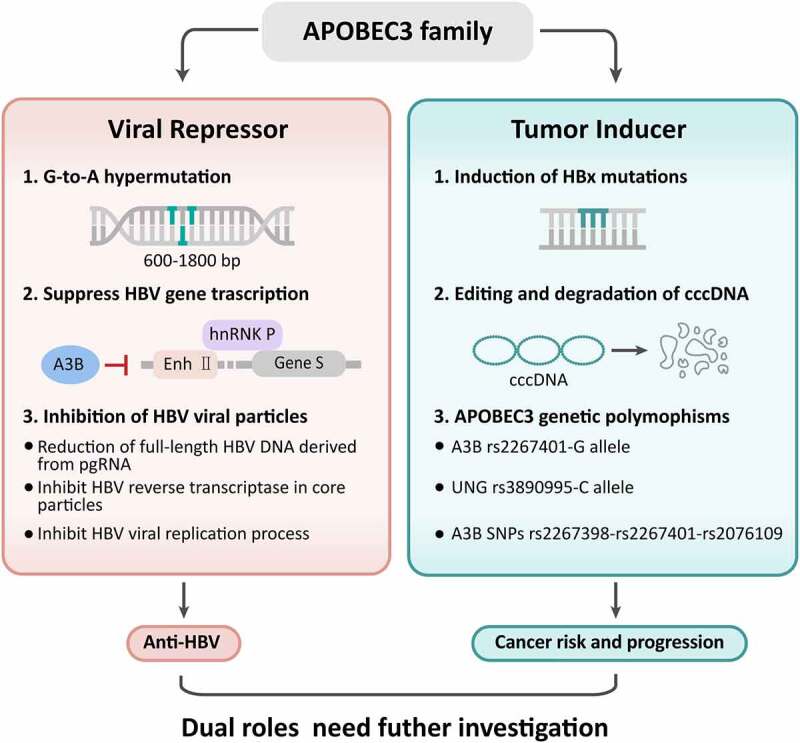

## 1. Introduction

Apolipoprotein B mRNA editing enzyme catalytic polypeptide-like (APOBEC) functions in the deamination of cytidine to uridine in DNA and RNA [1–3]. As a subfamily member, human APOBEC3 (A3) is composed of seven genes with one conserved zinc-coordinating domain (A3A, A3C, and A3H) or with double domains (A3B, A3D, A3F, and A3G) [4–6]. As a component of the human innate immune system, all A3 subfamily members serve as intrinsic defenses against both exogenous and endogenous retroviruses, including human immunodeficiency virus (HIV), herpesviruses, parvoviruses, papillomaviruses, retrotransposons, coronavirus, human papilloma virus, and HBV [7–15]. During the reverse transcription process, A3 deaminates cytidines in the intermediate of viral single-stranded DNA, and adenosines are transcribed from uridines, leading to G-to-A hypermutations in second-strand DNA synthesis [1,15–17]. The current consensus has demonstrated that A3 enzymes play a vital role in maintaining a variety of biochemical processes, such as the regulation of protein expression and innate immunity [3]. However, increasing evidence has indicated that the deaminated deoxycytidine biochemical activity of A3 proteins in single-stranded DNA makes them a double-edged sword. Under pathological conditions, aberrant expression of A3 genes with improper deaminase activity regulation could threaten genomic stability and eventually result in cancer [1–3,18,19].

All the members of the A3 family are located at 22q13.1. A3B localizes to the nucleus. A3A, A3C and A3H are distributed cell-wide and are able to enter the nucleus via passive diffusion. A3D, A3F and A3G are cytoplastic and can enter the nucleus through an active import mechanism [1,3,14]. In addition, A3A and A3G can be transiently recruited to the nucleus [20–23]. When misregulated, overexpression of A3 subunits has been described as the main endogenous source of mutations in several human malignancies, including breast, bladder, head and neck, ovary, cervical, ovarian, lung and liver cancer, as well as myeloma [22,24–30]. In addition, A3 members are frequently upregulated in HBV-infected hepatocytes [31], and upregulation of A3 genes has also been linked with poor survival of cancer patients [32,33].

Hence, understanding the antiviral roles and cancer-promotion functions of A3 genes in the HBV-infected population is highly important [34]. The aim of this review is to summarize the biochemical functions of A3 genes in HBV infection, discuss the underlying mechanisms of anti-HBV activities, and address the tumorigenic roles of these genes in hepatocarcinogenesis, in the hope to draw a diagram for outlining the influence of A3 genes in HBV infection and HBV-related tumorigenesis

## 2. A3 expression

### 2.1 A3 expression in chronic HBV infection

The mRNAs of A3B, A3C, A3F, and A3G are detectable in normal human liver tissues [35]. A3-footprinted genes were obtained by reverse transcribing HBV. NNGANN motif depletion has been observed for the C and preC/HBeAg coding sequences of HBV, supporting A3 editing activity on the DNA negative strand involvement in the reverse transcription process [10]. Compared to normal liver samples, the 5/7 A3 genes (A3B, A3C, A3D, A3G, and A3H) in liver cirrhosis samples were significantly upregulated in HCV/HBV- and HBV-infected cases [31]. Compared to healthy individuals, A3G mRNA levels were significantly downregulated in CHB patients, and A3G was significantly decreased in high-HBV load patients compared with low-HBV load cases [36]. Immunohistochemistry also verified that A3G was remarkably lower in CHB patients than in healthy controls [37]. In contrast, another report showed that A3G was upregulated in peripheral blood mononuclear cells (PBMCs) in CHB patients compared with healthy individuals [38]. Compared to the uninfected liver, A3B and A3G were remarkably increased in the chronically HBV-infected liver. Furthermore, there was a trend toward a positive correlation between HBV covalently closed circular DNA (cccDNA) levels and A3 gene expression, including A3A, A3B, and A3G [39]. During alanine aminotransferase (ALT) flare-up in HBV carriers, G-to-A hypermutation of HBV increased [40]. Moreover, A3G mRNA in liver tissues is moderately associated with the ALT content [41]. These controversial findings surrounding A3G levels in chronic HBV infection need further investigation with a large number of samples.

### 2.2 A3 expression in liver cancer

Multiple approaches confirmed that the content of A3G in liver tissues was the highest in CHB patients, slightly lower in patients with liver cirrhosis and the lowest in liver cancer [41]. No significant difference in liver A3G products was found between CHB patients and HCC patients in another report [38].

Since the G1896A pre-core (PC) and A1762T/G1764A basal core promoter (BCP) mutations are established risk factors for HCC [42], a recent report addressed A3 expression between HBV wild-type and HBV PC/BCP variants transfected HepG2 cells [43]. The presence of the viral DNA or different variants did not result in significant changes in A3 family mRNA induction. Immunoblot and densitometry analysis confirmed that the amounts of A3G protein were significantly increased in wild-type cells compared to mock and HBV X/BCP/PC variants. Differential DNA denaturation PCR (3D-PCR) indicated that no visible differences in DNA denaturation was observed in the cccDNA between the X/BCP/PC variants and wild-type HBV [43]. That is, differences in A3 expression between wild-type and mutant HBV do not result in alterations in HBV cccDNA G-to-A hypermutation in hepatoma cells [43]. . In our previous report, we found that A3B, A3D, A3F and A3H were overexpressed in tumor tissues compared to non-tumor tissues in HCC patients [32]. In another dataset, A3B showed higher level in HCC tumors and that in non-tumor samples, while A3A and A3C were downregulated in tumor samples in HCC patients [33]. In a study by Luo X et al, A3A, A3B and A3D were overexpressed in cancerous tissues compared to the contiguous noncancerous tissues, while A3C, A3E, A3F and A3G were downregulated in 49 HCC samples [44]. In 29 HCC and matched surrounding nontumor samples, an increased expression of A3G or A3F in HCC tumor tissues was observed in only a small fraction of the samples, while A3B transcripts were significantly elevated in 24 of 29 HCC samples [45]. Recently, our experimental analysis showed that A3F mRNA and A3F protein were also upregulated in tumor tissues compared to adjacent tissues in HCC patients [46].

## 3. Interferon (IFN) induction for A3 genes

IFN can induce A3 gene activation [47,48]. In primary human hepatocytes, IFNα stimulated the expression of A3C cytidine deaminase by up to 14-fold, and the mRNAs of A3G, A3F, and A3B reached expression levels of 10%, 3%, and 3%, respectively [35]. A3A, A3B, A3C, A3D, and A3G were all upregulated in CHB patients who received IFN treatment. A3A was significantly higher in IFN responders than in nonresponders. In HBV-infected cells, primary hepatocytes, and human liver tissues, activation of IFN could upregulate A3 clusters, including A3A, A3B, A3C, A3F, and A3G [23,35,49]. Human hepatocytes treated with IFN presented increased production of A3B, A3F and A3G [50]. IFN, but not entecavir, could significantly upregulate A3G levels in PMBCs of CHB patients [51,52]. However, virion infectivity factor (Vif)-induced A3G degradation did not eliminate the anti-HBV effects of IFN [52]. Additionally, IFN treatment could induce A3F and A3G mRNA expression and HBV cccDNA degradation in human hepatocytes [53]. Multiple inﬂammatory cytokines, including INFα, IFNγ, and tumor necrosis factor α (TNFα), promote nuclear A3 activation and cccDNA deprivation [49,54,55].

A3F mRNA was expressed in HepG2 and Hep3B cell lines and could be induced by IFNα in a dose- and time-dependent fashion [48]. In primary hepatocytes of several healthy donors, A3F was detectable, and A3F mRNA could be induced by IFNα in these hepatocytes. A3G induced by IFNα varies between approximately 5- and 19-fold in primary hepatocytes [48]. In macrophages, A3F and A3G could also be efficiently induced by IFNα from some healthy donors. These researchers also indicated that IFNα upregulated A3F and A3G in liver cell lines and primary hepatocytes but not in primary CD4^+^ T cells [48].

However, Turelli et al. revealed that A3 enzymes are not essential effectors of the anti-HBV activities of type I and II IFN. They found that murine A3 (muA3) can also inhibit HBV replication. Although the baseline expression of muA3 in mouse liver was low, it was evaluated under IFN induction. In contrast, in HBV-transgenic muA3 knockout mice, IFN-induced blockade of HBV DNA production was as effective as that in control HBV-transgenic muA3-competent animals [56]. A study by Jost et al. demonstrated that downregulation of A3B, A3F, and A3G by RNA interference does not neutralize the inhibitory effect of IFNs on HBV, suggesting that these editing enzymes are not indispensable effectors of IFN in its anti-HBV activity [50]. Inconsistent with previous reports [23,35,49], Koh et al. also identified that IFNα treatment for 24 to 48 hours decreased A3A mRNA and caused a less robust increase in A3B and A3G mRNA [57].

## 4. Anti-HBV activities of A3 genes

More evidence has demonstrated that HBV DNA is vulnerable to A3 cytidine deaminase genetic editing [31,58,59]. A3G, which can edit up to 35% of the genome of HBV, is the most powerful inhibitor of HBV replication [31,60]. Both in vivo and in vitro, A3 clusters could significantly inhibit the levels of viral parameters, including HBsAg, HBeAg, HBcAg, HBV DNA, HBV cccDNA and HBV RNA [54,61–65]. A3 gene levels at IFN treatment endpoints are partially associated with the corresponding absolute DNA level, HBsAg degree and HBV DNA decline [66,67]. In transfection, the full-length protein A3B(L), as well as A3G and A3F, inhibited HBV replication efficiently in vitro, whereas the truncated splice variants A3B(S) and A3C had no effect. A3G, A3F, and A3B(L), but not A3B(S), induced massive G-to-A hypermutations in a small portion of the replicated HBV genomes. These results suggested that the editing enzymes A3B(L), A3F, and A3G, which are expressed in the liver and upregulated by IFNα in hepatocytes, are candidates that contribute to HBV clearance [35]. G-to-A hypermutated HBV genomes were recovered from transfection experiments involving A3B, A3C, A3F, and A3G, indicating that all four enzymes were able to extensively deaminate cytidine residues in minus-strand DNA. In addition, A3B, A3F, and A3G deaminated HBV plus-strand DNA, as well. From the serum of two out of four patients with high HBV viremia, G-to-A hypermutated genomes were recovered, suggesting that G-to-Aa hypermutations induced by A3 clusters are part of the natural cycle of HBV infection. These findings demonstrated that human A3 enzymes can impact HBV replication via cytidine deamination [65].

In Huh-7 and HepG2 cells, as well as the avian hepatoma cell line LMH, A3G blocked replicative HBV intermediates in a dose-dependent manner. Even a single N-terminal or C-terminal cytosine deaminase domain could inhibit HBV replication in HepG2 and Huh7 cells [68]. However, transient A3A expression does not cause a decrease in core-associated HBV DNA in HepG2 cells, and A3A overexpression does not decrease HBV replicative intermediates, even though it can increase the hypermutation of HBV genomes in a dose-dependent manner. This indicates that A3A has a negligible anti-HBV effect, although it induces hypermutation of HBV genomes [58].

Higher anti-HBV activity of the A3C I188 variant for HBV DNA was observed compared to that of the A3C S188 variant. Among A3H haplotypes and splicing variants, hap II SV-183 caused the most significant decrease in HBV DNA levels, while only a small amount of decrease was detected in hap III, hap IV, and SV-154 (with hap II) transfectants. No significance of HBV DNA was found between A3G H186 and R186 [69]. The present A3C could be easily packaged into replication-competent capsids and majorly deaminated extensive G-to-A mutations of newly synthesized HBV DNA genomes, while no A3C-induced HBV DNA mutations were detected, suggesting that A3C could account for innate anti-HBV host responses [64].

Single-nucleotide polymorphism (SNP) analysis revealed that both the C and G alleles of rs3077 and rs9277535 significantly increased the risk for advanced liver disease in HBV carriers without the A3G H186R variant [70]. In a cohort study including 179 chronic HBV carriers and 216 healthy controls, no significance of the frequencies of deleted A3B alleles or genotypes was found between CHB patients and healthy controls. However, the progression of liver disease is remarkably faster in individuals with the A3B-deleted genotype than in insertion subjects. Analysis of the A3G H186R polymorphism showed that there was no significant difference in R/R genotype frequencies between HBV-infected patients and healthy individuals. Amplified virus fragments showed singlet G-to-A transitions highly reminiscent of A3G activity. Interestingly, the HBV virus loads in hemi/homozygous carriers with A3B deletion were significantly lower than those in wild-type subjects [71].

In a quail cell line, a 3D PCR approach showed that all genes in the A3 family except A3D could deaminate HBV DNA at 10^−2^ to 10^−5^ levels in vitro, which proved that A3A was the most effective editor [72]. In contrast, another report revealed that A3D binds to A3F and A3G, and A3D facilitates HBV replication by inhibiting A3F and A3G from HBV particles. A3D is capable of hyperediting HBV DNA, and upregulation of A3D results in higher HBV DNA levels. Moreover, as a part of the innate restriction factor, A3D serves as a proviral phenotype [9]. A3F could also inhibit HBVDNA levels but to a lesser extent compared to A3G. No RNA editing was detected in the case of A3F, and infrequent DNA editing activity of A3F was also observed, suggesting that A3F-mediated hyperediting does not seem to be a promising innate defense mechanism for hepadnaviruses [73].

## 5. Anti-HBV mechanisms of A3 genes

**Figure 1.**
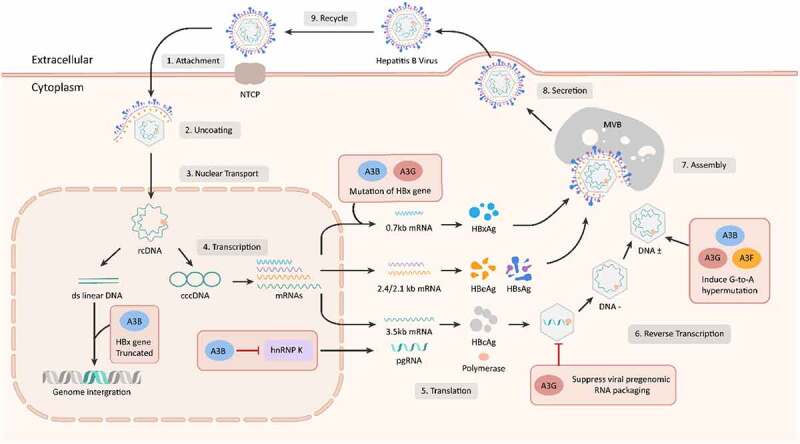
The process of HBV infecting liver cells and mechanisms of A3 genes in anti-HBV

A3 genes influence HBV replication through various modes including mediation of G-to-A hypermutation, suppress HBV gene transcription, and inhibition of HBV viral particles. The HBV life cycle and potential mechanisms of A3 gene in anti-HBV process were illustrated in Figure 1.

### 5.1 G-to-A hypermutation

G-to-A hypermutation exists in HBV isolates and influences HBV pathogenesis [74,75]. Genome-wide ultradeep pyrosequencing revealed that G-to-A hypermutation mainly occurred in a region between nucleotide positions 600 and 1800, which is usually single-stranded in mature HBV particles. The rate of hypermutation in HBeAg-negative patients was more than 10-fold higher than that in HBeAg-positive patients. A higher hypermutation rate was significantly correlated with the degree of fibrosis in the HBeAg-negative population. High hypermutation rates of HBeAg-positive chronic hepatitis patients were significantly associated with the relative prevalence of the G1764A mutation [76]. Specific changes from G-to-A result in HBeAg-negative HBV variants, which are usually isolated from acute fulminant hepatitis and HBV vaccine escape mutants [63]. A study by Noguchi et al. showed that HBV DNA hypermutation was detected in 1 of 8 acute HBV-infected patients and 4 of 10 CHB patients who were eAb-positive. Up to 72.5% of G residues were mutated in the hypermutated clones. G-to-A mutated HBV DNA genomes were observed in HepG2-derived cell lines, which can continuously release HBV into the supernatant [74]. In Huh-7 transfectants, all tested A3 variants, including A3C S188, A3C I188, A3G H186, A3G R186, A3H hap II, hap V, hap VI, and hap VII, exhibited hypermutation activity. Among these variants, A3C I188 showed the most powerful anti-HBV activity. Sequencing analysis shows that a 1.8-fold higher mutation frequency in HBV DNA was detected from A3C I188 compared to S188. On average, 8.4 G-to-A mutations per clone were identiﬁed within the small HBV X gene region (694 base pairs) of A3C I188 transfectants, indicating that the higher hypermutation activity of the A3C I188 variant reﬂected its higher anti-HBV activity [69]. However, A3G inhibits HBV replication by suppressing viral pregenomic RNA packaging, rather than by inducing G-to-A hypermutation [63]. Although some reports revealed that HBV DNA species show G-to-A hypermutation via A3 family members, a study by Lau et al. indicated that no visible difference in HBV DNA denaturation was found in cccDNA one day posttransfection between the HBV X/BCP/PC variants and wild type [43]. Recent evidence has revealed that A3F can inhibit HBV replication with no or very little G-to-A hypermutation [65,73]. The roles and mechanisms of G-to-A hypermutation in suppressing HBV replication need further investigation to uncover the anti-HBV defense system.

### 5.2 Interaction with HBV protein

The interaction of the HBV core protein with nuclear cccDNA results in cytidine deamination, purine/pyrimidine site formation, and cccDNA degradation, which finally prevents HBV reactivation [49,54]. A3B has been observed to suppress HBV in both HBsAg, HBeAg and HBV core-associated DNA synthesis. The A3B protein inhibited the binding of heterogeneous nuclear ribonucleoprotein K (hnRNP K), which is a positive regulator of HBV expression, to enhancer II of HBV (Enh II) and S gene transcription of HBV. In addition, A3B directly inhibited HBV S gene promoter activity. All these findings demonstrated that upregulation of A3B might be effective for HBV clearance in vivo [77].

Early steps of HBV viral morphogenesis, including RNA and protein synthesis, binding of pregenomic RNA to core proteins, and self-assembly of viral core proteins, were unaffected by A3G. The total HBV RNA production was not reduced by A3G, while pgRNA coprecipitated with cytoplasmic core proteins independently of the absence of A3G. In addition, viral DNA products were similar in the absence and presence of A3G. Notably, the presence of A3G was linked to the reduction in full-length HBV DNA derived from pgRNA. The anti-HBV effect of A3G was not altered by abolishing or enhancing the expression of the accessory HBV X protein [73]. This study was unable to detect the reduction of encapsidated pregenomic RNA in HBV core particles, and reported that the reverse transcription of HBV cDNA in core particles did not alter in the presence of A3G. The unusual sensitivity of core particles to micrococcal nuclease might due to unstable core structure [78]. In a report by Nguyen et al, A3G was cotransfected with an HBV mutation within the ribonuclease-H-domain of the HBV polymerase. They found that A3G had little effect on the packaging of pregenomic RNA within HBV core particles, blocked early steps in viral minus-strand DNA synthesis and did not enhance HBV DNA degradation or block late stages of DNA synthesis [79]. Notably, A3G had a profound suppress of HBV reverse transcriptase within core particles by A3G proteins [78,79]. Moreover, A3G mediated inhibition of HBV viral replication process including reduction of nucleocapsid DNA, pre-C mRNA, and secreted viral particle-associated DNA [80]. In another report, A3B, A3C, A3F, and A3G proteins could be immunoprecipitated with antibodies against HBV core protein, indicating physical interaction of these proteins with HBV core particles [35].

In HepG2 cells, approximately 85.2% of cells coexpressed A3G and HBV core protein (HBc), and more extensive colocalization of A3G and HBc was found in a number of coexpressing cells. An interaction between A3G and HBc was also detected in living cells by a fluorescence resonance energy transfer approach. A surface plasmon resonance assay tested a direct interaction between A3G and HBc, and RNA had no impact on stabilizing the interaction [81]. In another report, A3G did not alter the formation pattern of HBc, as verified by velocity sedimentation gradient analysis and native agarose analysis. No direct association of A3G with HBc was found in immunoprecipitation experiments.

A study by Koh et al. indicated that HBV-speciﬁc TCR-reprogrammed resting T cell treatment for 24 hours or 48 hours could induce a 20% to 40% increase in A3A and A3B mRNA, respectively, and a 450% increase in A3G mRNA in HepG2 cells. These data suggest that HBV-speciﬁc TCR-reprogrammed resting T cells might be capable of rapidly activating the A3 innate antiviral pathway by interacting with the lymphotoxin-β receptor on HBV-infected targets. Lymphotoxin-β increased by 9-fold and 19-fold with 0.5 million and 1 million resting T cells, respectively. A3B mRNA increased by 45-fold in mice treated with 1 million resting T cells. The authors concluded that resting T cells activate lymphotoxin-β and A3B in HBV-infected primary hepatocytes and inhibit HBV replication without inducing liver inflammation [57].

## 6. A3 and hepatocellular carcinoma (HCC)

A3 cytidine deaminase activation boosts the immune response by mutating immune or HBV viral genomes. Because of their genome-mutating activities, A3 genes are also drivers of tumorigenesis [3,25,82–84]. Upregulation of A3 genes has been found in many human malignancies [29,32,33]. The A3 genes can catalyze clustered hypermutation in cancer cell genomes; however, A3-catalyzed mutation clusters only account for 10% or less of the total mutations [85,86]. Consequently, the A3 genes can catalyze modest changes in the cancer genome to promote tumor evolution [87]. In the last few years, a role in cancer initiation has also emerged for the A3 subbranch of the family, for example, A3A [88], A3B [29,30], A3F [32,33], and A3H [22]. Higher levels of A3 expression were correlated with decreased survival rates in human cancers, including breast cancer [89,90] and HCC [32,33]. Mutagenic effects induced by A3 DNA dC->dU-editing enzymes also account for chemotherapy resistance in urothelial carcinoma [91]. In addition, a meta-analysis also indicated that A3 deletion accounts for increased susceptibility to human cancers [92]. Many reviews have summarized the roles of the A3 subbranch in the development of human cancers [3,24,29,83,84,87], while the impact of A3 genes on HCC tumorigenesis and aggressiveness has not been well elucidated. The studies investigating associations between A3 genes and HCC have been summarized in Table 1.

**Table 1.** Studies investigated A3 genes and HCC development

Author	Year	A3 genes	Cell lines	Participants	Main findings	Reference
He X, et al	2019	A3A, A3B, A3H	/	HBV-related HCC: 285; CHB: 104; HBV-related cirrhosis: 265	No relationship showed between A3A, A3B and A3H SNPs and CHB progression or HCC development.	[26]
Yang Z, et al	2015	A3A, A3B, A3C, A3D, A3F, A3G, A3H	/	HCC: 240	A3G and A3F were risk factors for HCC progression and survival; A3C and A3H might play favorable roles in HCC aggressineness and survival.	[32]
Yang Z, et al	2015	A3A, A3B, A3C, A3F, A3G	/	HBV-related HCC: 220	A3F was a risk facor for HBV-related HCC recurrence.	[33]
Luo X, et al	2016	A3A, A3B, A3C, A3D, A3F, A3G, A3H	/	HCC: 49	A3B contributed to cccDNA editing and subsequent degradation in cancerous tissues.	[44]
Xu R, et al	2007	A3A, A3B, A3C, A3G, A3F	HepG2	HCC: 29	A3B promoted the growth of neoplastic human HepG2 liver cells and up-regulated heat shock transcription factor1 (HSF1) expression; Some A3 genes (A3B, A3C, A3F and A3G) play a role in the carcinogenesis of HCC through the generation of HBx mutants.	[45]
Yang Z, et al	2018	A3F	SK-Hep1; Bel-7404	HCC: 8	A3F was upregulated in tumor samples and promoted cell proliferation and migration.	[46]
Liu W, et al	2019	A3B	/	Health controls: 1449; HBV clearence subjects: 300; ASC: 511; CHB: 1016; HBV-related cirrhosis: 674; HBV-related HCC: 1271	A3B rs2267401-G and UNG rs3890995-C allele were signiﬁcantly increased HCC risk. A3B rs2267401-GG genotype, higher A3B, and higher A3B/UNG expression ratio predicted poor HCC prognosis.	[93]

ASC, asymptomatic hepatitis B virus carriers; CHB, chronic hepatitis B; HCC, hepatocellular carcinoma; SNPs, single nucleotide polymorphisms.

In a study including 35 matched tumor and nontumor tissues from HCC patients, sequence analysis showed that G-to-A hypermutation was significantly higher in HBV cccDNA in tumor samples than in nontumor samples. The frequency of G-to-A mutations was 4.85 per 1000 sites in tumor samples, while it was only 0.16 in nontumor samples. Among the A3 subbranches, A3B was significantly upregulated at both the transcriptional and protein levels in tumor samples compared to nontumor samples. These findings suggest that upregulation of A3B contributes to editing and degradation of cccDNA in HCC patients [44]. Xu et al. also indicated that A3-mediated HBx mutations, especially C-terminally truncated mutants, enhanced the colony-forming ability and proliferative capacity of neoplastic cells. G-to-A hypermutation-mediated HBV X mutants were also observed in preneoplastic liver samples from active CHB patients. Consistent with a previous report [44], A3B was significantly upregulated in the tumor tissues of HCC patients. In addition, A3B overexpression promoted the proliferation of neoplastic human HepG2 liver cells. They concluded that some of the A3 deaminases play a role in the carcinogenesis of HCC by generating HBx mutants, thus providing selective clonal growth advantages for preneoplastic and neoplastic hepatocytes [45].

A study by He et al. enrolled 104 CHB patients, 265 HBV-related cirrhosis patients and 285 HBV-related HCC patients. single-nucleotide polymorphisms (SNPs) including two A3A rs7286317 and rs7290153, A3B rs2267398, rs2267401 and rs2076109, and A3H rs56695217, rs139302, rs139297, rs139316 and rs139292 were genotyped. No significant correlations between these SNPs and the development of HCC were found. However, mutant haplotypes C-T-A, C-T-G, T-G-G and T-T-G from the A3B SNPs rs2267398-rs2267401-rs2076109 carried a lower risk of HCC compared to the reference haplotype [26]. Since A3-UNG imbalance contributes to somatic mutations, Liu et al. detected A3 promoter and UNG enhancer genetic polymorphisms and determined HBV mutations in 5621 participants. They found that the A3B rs2267401-G allele and UNG rs3890995-C allele significantly increased the HCC risk. The rs2267401-G allele was significantly associated with generation of the APOBEC signature HBV mutation, whose frequency consecutively increased from asymptomatic HBV carriers to HCC patients. The interaction of the rs2267401-G allele with the rs3890995-C allele also increased the HCC risk. The A3B rs2267401-GG genotype, higher A3B expression, and higher A3B/UNG expression ratio in HCCs indicated a poor prognosis. APOBEC signature somatic mutation predicts poor prognosis in HBV-free HCCs rather than in HBV-positive HCCs [93].

Previously, we compared A3 gene expression between tumor and nontumor tissues in HCC patients in a public database from the Gene Expression Omnibus (GEO). In GSE36376, A3B, A3D, A3F and A3H were upregulated in HCC tumor tissues compared to nontumor tissues. High levels of A3G and A3F contributed to poor overall survival (OS) and disease-free survival (DFS), respectively. In contrast, A3C and A3H overexpression was associated with favorable OS and DFS, respectively. In addition, A3F upregulation might increase the risk of vascular invasion, intrahepatic metastasis, and alpha-fetoprotein elevation, while high A3H decreased these risks [32]. In another GEO profile, GSE14520, which is upregulated in tumor tissues, A3B was negatively associated with OS in HBV-related HCC patients. A3F expression in tumor tissues more likely coexists with multinodular tumors than those with low A3F levels. High levels of A3F in tumor tissues might account for recurrence in HBV-related HCC patients [33]. Similarly, we also detected that A3F was significantly overexpressed in tumor tissues compared to nontumor tissues in HCC patients in both the TCGA dataset and HCC samples from our own institute. In vitro, A3F enhanced cell growth ability and migration in the human liver cancer cell lines SK-Hep1 and Bel-7404. A3F treated with RNA interference resulted in decreased levels of key molecules in the intestinal immune network for the IgA production signaling pathway, including CCR9, CCR10, CXCR10, and polymeric immunoglobulin receptor (pIgR), in both SK-Hep1 and Bel-7404 cells [46].

## 7. Conclusion and future prospects

A3 family members exhibit effective antiviral activities against HBV infection. In HBV infections, A3 genes exert anti-HBV roles by inducing G-to-A hypermutations and interactions with HBV proteins and are active molecules induced by interferon and other cytokines. On the other hand, A3 enzymes were shown to induce tumor mutations through aberrant DNA editing mechanisms. In HCC tumorigenesis, A3-mutated HBV X gene mutants and A3-induced HBV genetic polymorphisms lead to the development of HBV-related liver cancer. That is, A3 genes are the underlying molecules in the pathogenesis of HCC. However, the expression of A3 genes in liver tissues of different stages of liver diseases is still controversial. The anti-HBV mechanisms and activities of A3 family members need further investigation in detail. Moreover, as a viral repressor and tumor inducer, dual roles of A3 genes in the development of HBV-related HCC are questionable. As we know, suppressing of HBV transcription and replication leading to lower risk of cancer occurrence and tumor aggressiveness, and HBV gene mutations contribute to higher risk of cancer development. Since members of A3 family own functional roles in both sides, the underlying links between anti-HBV and tumor-promotion of A3 genes should be addressed. Which function of A3 is dominant is still a question in the development of HBV-related liver cancer. Conclusively, the potential mechanisms need to be investigated in different populations to understand their true association with cancer risk, viral restriction, and mutagenesis.

## Data Availability

Not applicable.

## Highlights

- A3 genes have universal anti-HBV activity.
- A3 inhibit HBV replication mainly through G-to-A hypermutations and suppress of HBV proteins.
- A3 induced genome mutations and A3 genetic polymorphisms contribute to hepatocarcinogenesis.
